# Kystes hydatiques cérébraux de l’enfant: à propos de 15 cas

**DOI:** 10.11604/pamj.2017.26.205.8398

**Published:** 2017-04-13

**Authors:** Abderrazzak El Saqui, Mohamed Aggouri, Mohamed Benzagmout, Khalid Chakour, Mohamed El Faiz Chaoui

**Affiliations:** 1 Service de Neurochirurgie, CHU Hassan II, Fès, Maroc

**Keywords:** Kyste hydatique, localisation cérébrale, chirurgie, TDM, IRM, Hydatique cyst, cerebral location, surgery, CT scan, MRI

## Abstract

L’objectif de notre étude est d’illustrer l’intérêt de l’imagerie en coupe (TDM, IRM) dans le diagnostic positif et le suivi post-thérapeutique du kyste hydatique cérébral chez l’enfant et de mettre en lumière les particularités et les difficultés rencontrées dans la prise en charge de la localisation cérébrale de cette affection, par notre expérience basée sur 15 cas de kyste hydatique cérébral de l’enfant. Il s’agit d’une étude rétrospective de 15 cas d’hydatidose cérébrale de l’enfant colligés sur une période de 10 ans. La TDM cérébrale en coupes axiales de 5 mm d’épaisseur sans et avec injection de produit de contraste a été réalisée chez 15 patients. L’IRM encéphalique a été réalisée en séquences pondérées en T1 et en T2 chez un patient dans les trois plans de l’espace sans injection de Gadolinium. L’âge moyen de nos patients était de 9 ans. La symptomatologie clinique était dominée par le syndrome d’hypertension intracrânienne. Le kyste hydatique était solitaire et se situait au niveau de l’étage sus-tentoriel avec un important effet de masse sur le système ventriculaire et la ligne médiane dans la majorité des cas. Tous nos patients ont été opérés et l’évolution était favorable dans tous les cas. La TDM représente l’examen de choix pour le diagnostic et le suivi postopératoire du kyste hydatique cérébral. L’IRM trouve son intérêt essentiellement dans le diagnostic des formes multiples et des formes atypiques permettant une planification thérapeutique plus adaptée.

Our study aimed to highlight the role of cross sectional imaging techniques (CT, MRI) in positive diagnosis and post-therapeutic follow-up of cerebral hydatid cysts in children as well as to describe the peculiarities and the difficulties encountered in the management of these cysts based on our experience about 15 cases. We conducted a retrospective study of 15 cases of cerebral hydatidosis in children whose data were collected over a period of 10 years. CT scan of the brain with 5 mm slice thickness without and with injection of contrast product was performed in 15 patients. One patient underwent brain MRI creating either T1-weighted or T2-weighted images in all three planes without Gadolinium injection. The average age of patients was 9 years. Clinical symptoms were dominated by intracranial hypertension syndrome. Hydatid cyst was solitary and was located in the supratentorial level with an important mass effect on the ventricular system and the median line in most cases. All patients underwent surgery and patients’ evolution was favorable in all cases. CT scan is the test of choice for the diagnosis and the postoperative follow-up of patients with cerebral hydatid cysts. MRI is used essentially in the diagnosis of multiple and atypical type of cerebral hydatid cysts, enabling the design of more effective treatment strategy.

## Introduction

La pathologie hydatique est très fréquente au Maroc et au pourtour méditerranéen [1]. La localisation cérébrale est rare, ne représente que 2% environ de toutes les localisations hydatiques de l´organisme et touche surtout l'enfant et l´adolescent [2]. Les signes cliniques sont ceux d'un processus intracrânien, la TDM souvent suffisante pour poser le diagnostic et le traitement reste chirurgical.

## Méthodes

Nous avons conduit une étude rétrospective, étendue sur 10 ans, portant sur les dossiers médicaux de 15 cas de kystes hydatiques cérébraux opérés au service de neurochirurgien du CHU Hassan II Fès-Maroc depuis janvier 2001 jusqu'au décembre 2011.

## Résultats

**Figure 1:**
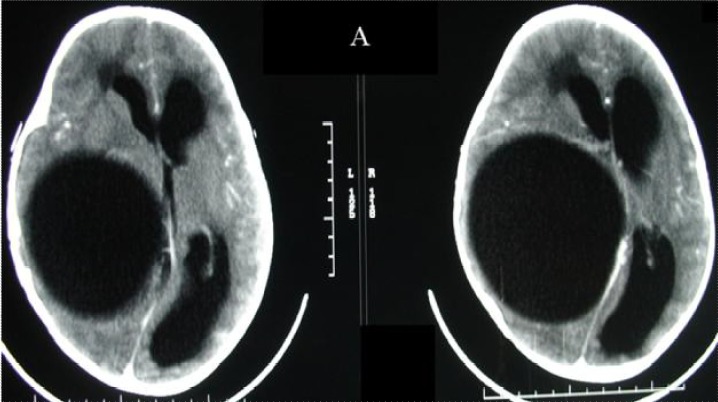
TDM cérébrale en coupes axiales avec injection de PDC montrant une volumineuse lésion fronto-temporo-pariétale droite, bien limitée, de même densité que le LCR, exerçant un effet de masse sur le ventricule latéral droit et la ligne médiane, sans oedème périlésionnel ni prise de contraste périphérique: aspect pathognomonique d’un KHC

**Figure 2:**
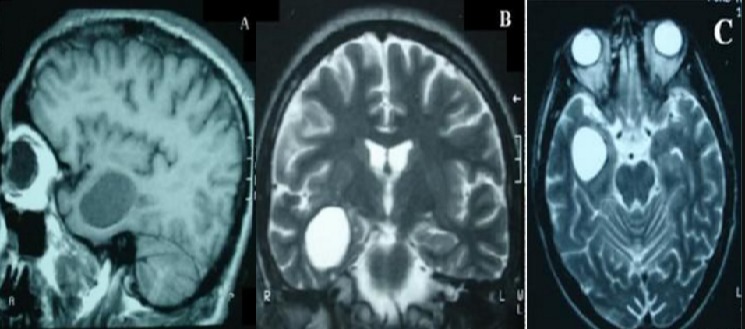
IRM cérébrale en coupe sagittale (A), coronale (B) et axiale (C) montrant une lésion bien limitée, qui apparaît en hyposignal sur les séquences pondérées T1(A) et hypersignal sur les séquences pondérées T2 (B et C) en faveur d’un KHC temporal droit

**Figure 3:**
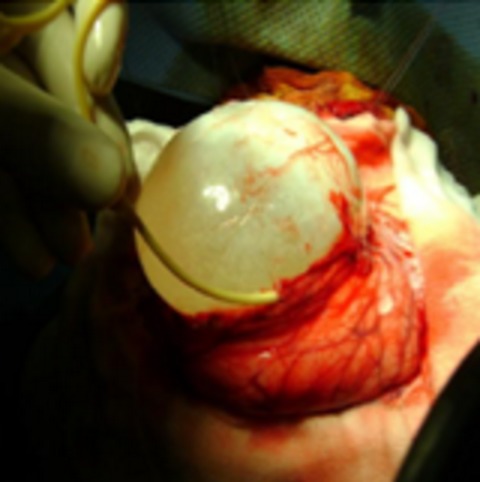
Glissement d’un cathéter entre le parenchyme cérébral et la paroi du kyste et accouchement du kyste par injection du sérum salé

L’âge moyen de nos patients était de 9 ans, avec des extrêmes allant de 3 à 14 ans, avec un sex-ratio de 1,5. Tous nos patients avaient une origine rurale et avaient un contact permanent avec les chiens. Le délai moyen du diagnostic était de 4 mois avec des extrêmes de 1 mois et 2 ans. La symptomatologie clinique était dominée par le syndrome d’hypertension intracrânienne qui a été retrouvé chez 14 patients, soit 93% des cas, 1 cas avait une cécité bilatérale, 8 autres cas présentaient un syndrome déficitaire type hémiparésie et chez 2 autres patients une macrocranie a été notée. Le fond d’œil, réaliser chez tous nos malades, a montré une atrophie optique chez un patient et un œdème papillaire chez un patient. Il était normal dans le reste des cas. La TDM cérébrale a été réalisée chez tous les patients; tous les kystes étaient solitaires, se situaient au niveau de l’étage sus tentoriel chez 14 patients, avec une prédominance de la localisation fronto-pariétale qui représentait 67% des cas. Le kyste se présentait sous forme d’une image intracérébrale hypodense bien limitée, arrondie ou ovalaire, de grande taille dans la majorité des cas avec une moyenne de 60 × 50 mm. Le kyste était uniloculaire dans 15 cas (100 %). La densité du kyste était identique à celle du liquide céphalo-rachidien, sans prise de contraste, ni œdème péri-lésionnel. Un effet de masse sur le système ventriculaire et la ligne médiane a été retrouvé dans 93,3% des cas (Figure 1) et une hydrocéphalie triventriculaire dans un seul cas. La paroi et les cloisons ne se rehaussaient pas après injection de produit de contraste iodé L’IRM encéphalique a été réalisée chez un patient. Elle a mis en évidence une formation kystique simple, hypo-intense en séquence pondérée en T1, hyper intense en séquence pondérée en T2, à contenu homogène et à contours nets, siégeant au niveau de la région temporal droit et n’exerçant pas d’effet de masse sur le système ventriculaire et la ligne médiane (Figure 2). Une échographie abdominale et une radiographie du thorax ont été demandées systématiquement chez tous nos patients, dans le cadre du bilan d’extension à la recherche d’autres localisations hydatiques, ils ont objectivé une localisation hépatique du kyste hydatique dans un cas. Tous nos patients ont été opérés et le geste chirurgical a consisté en l’accouchement du kyste (Figure 3) par hydrodissection utilisant le sérum salé hypertonique selon la technique d’Arana Iniguez avec des suites simples. L’évolution était favorable chez 14 patients. Cependant le patient présentant une atrophie optique bilatérale a gardé une cécité binoculaire séquellaire.

## Discussion

La maladie hydatique est une affection parasitaire qui constitue un problème de santé publique dans de nombreux pays d’élevage traditionnel du bétail, dont le Maroc, où elle sévit à l’état endémique [1]. Elle est secondaire à l’infestation de l’organisme par l’embryon hexacanthe de l’Echinococcus granulosus [1, 2]. Le bassin méditerranéen présente actuellement le plus grand foyer mondial d’hydatidose, son indice d’infestation dans les pays en voie de développement varie entre 5 et 18 cas/100 000 habitants par an [3]. Les localisations fréquemment rencontrées sont de siège hépatique (48 %) et pulmonaire (36 %) alors que la localisation cérébrale est rare et n’excède pas 2% des cas [2, 3]. La rareté de la localisation cérébrale de l’échinococcose peut s’expliquer par le passage du parasite par deux filtres (hépatique puis pulmonaire) avant d’atteindre la grande circulation [1]. Elle est plus fréquente chez l’enfant et l’adulte jeune (50 à 70 % des cas) [3] et survient le plus souvent avant l’âge de 15 ans. Cependant, toutes les tranches d’âge peuvent être affectées [3]. La progression clinique de la maladie chez les adultes est plus rapide que chez l’enfant, ceci s’explique par l’inextensibilité de la boîte crânienne chez l’adulte. Le tableau clinique est dominé par l’hypertension intracrânienne, les troubles visuels et les syndromes déficitaires focaux [2, 3]. La TDM cérébrale reste l’examen de choix et de première intention dans le diagnostic du kyste hydatique cérébral, elle permet de préciser le siège, le nombre, le volume et le contenu du kyste ainsi que ses rapports avec les structures voisines aidant ainsi au choix de la voie d’abord chirurgicale [1, 3].

Dans la majorité des cas, l’aspect scanographique est typique sous forme d’une formation unique, sphérique ou ovalaire, située en plein parenchyme cérébral, de grande taille, à paroi fine et régulière, tracée au compas et ayant la même densité que le liquide céphalo-rachidien (LCR); elle exerce un effet de masse sur les structures médianes et les ventricules latéraux sans prise de contraste et sans œdème péri-lésionnel [1, 2]. L’absence quasi-constante de rehaussement périphérique par le produit de contraste s’explique par la minceur du périkyste au niveau du cerveau et de la membrane hydatique adhérente à ce périkyste [1]. Il n’est pas possible de distinguer en TDM entre péri kyste et membranes kystiques sauf si, exceptionnellement, des fragments de membrane proligère deviennent flottants [4] lors de la fissuration du kyste. D’un autre coté le passage de liquide hydatique dans le tissu cérébral va être à l’origine d’une réaction inflammatoire et d’un épaississement du périkyste. Ainsi œdème et prise de contraste, quand ils existent, signent l’insuffisance d’étanchéité liée à la fissuration du kyste [1, 5]. Les localisations cérébrales sont généralement hémisphériques sus-tentorielles et sous-corticales particulièrement dans le territoire de l’artère cérébrale moyenne au niveau du lobe pariétal [5], avec un cortex laminé et pratiquement sans substance grise repérable entre la voûte et la paroi du kyste. De rares cas de localisations intraventriculaires et au niveau de la fosse postérieure ont été rapportés [6, 7]. Concernant notre série, les kystes se situaient au niveau de l’étage sus-tentoriel chez 14 patients avec un cas au niveau de la fosse cérébrale postérieur. Le kyste hydatique cérébral siège le plus souvent à gauche (c’est le cas de 10 de nos patients), en effet, la naissance directe de la carotide commune gauche de l’aorte rend plus aisé la migration directe de l’embole vers le cerveau [1, 7]. Le kyste hydatique cérébral est souvent unique, (dans notre étude, tous les kystes étaient solitaires); les localisations multiples sont rares et sont généralement la conséquence d’une rupture spontanée ou peropératoire et parfois d’une embolisation massive à partir d’un kyste rompu dans le ventricule gauche [1, 3]. Les calcifications sont extrêmement rares, inférieures à 1% [8]. Les déformations osseuses rencontrées chez l’enfant à type d’amincissement de la voûte et de disjonction des sutures sont le corollaire de la surprenante tolérance liée à l’extensibilité de la boîte crânienne chez l’enfant. La localisation cérébrale est dans 10% des cas associée à d’autres localisations viscérales notamment pulmonaire et hépatique [5, 7]. Ces localisations doivent être systématiquement recherchées par la radiographie pulmonaire et l’échographie abdominale [3, 8]; dans notre série, une localisation hépatique du kyste hydatique a été retrouvée chez un patient.

Devant ces aspects typiques, il est généralement facile d’éliminer d’autres pathologies. Le diagnostic différentiel peut se poser avec certaines lésions kystiques, en particulier le kyste arachnoïdien, épidermoïde, l’astrocytome kystique, le craniopharyngiome et l’abcès du cerveau mais en région endémique, le diagnostic de kyste hydatique est évoqué d’emblée [1]. L’IRM offre actuellement non seulement des informations diagnostiques supplémentaires de la maladie hydatique cérébrale mais permet surtout une planification thérapeutique plus adaptée. Elle met en évidence une formation liquidienne sphérique, de contours fins, contenant un liquide ayant les mêmes caractéristiques en imagerie que le LCR [3]: hypointense en séquence T1 et hyperintense en séquence T2 avec une très fine paroi (périkyste) en hypersignal relatif T1 et hyposignal T2 caractéristique [9, 10]. L’annulation du signal sur les séquences Flair et l’hyposignal franc en diffusion caractérise également le kyste hydatique. L’hypersignal relatif de certains contenus kystiques en T1 serait lié à l’existence de sable hydatique. L’absence d’œdème péri-lésionnel et de prise de contraste des kystes non compliqués est encore plus patente en IRM [1]. Ainsi l’IRM démontre les caractéristiques de signal du kyste hydatique et les éventuelles adhérences que le périkyste peut avoir avec les structures avoisinantes, élément très important dans la planification de l’acte chirurgical prévenant la rupture accidentelle [1]. Les études spectroscopiques par résonance magnétique (SRM) du kyste hydatique sont très rares et démontrent un profil métabolique différent de ceux des autres lésions kystiques cérébrales avec l’existence en intrakystique de pics de lactate, alanine et pyruvate. Ces mêmes métabolites sont également retrouvés dans les kystes cysticercosiques avec prédominance du pic de pyruvate dans le kyste hydatique. Ce métabolite apparaît comme un potentiel marqueur de l’étiologie parasitaire et peut être même de la viabilité des kystes [11, 12]. Ainsi la SRM apporterait un argument diagnostique supplémentaire très utile en cas de problèmes de diagnostic différentiel. Elle jouerait aussi un rôle dans le monitoring des lésions résiduelles ou des récidives sous traitement médical [13].

L’IRM est meilleure également dans la détection des kystes hydatiques cérébraux multiples qui sont très rares (aucun cas dans notre série). Ils sont le résultat soit d’une rupture spontanée, traumatique ou peropératoire d’un kyste hydatique solitaire du cerveau soit d’une rupture d’un kyste cardiaque intraventriculaire gauche. Les kystes multiples sont alors de taille plus petite, multi ou uni-vésiculaires et disséminés dans les deux hémisphères cérébraux [1]. Enfin, l’IRM définit mieux les rapports de la lésion avec les structures avoisinantes ce qui aide également à la planification chirurgicale [14]. Le bilan biologique est non spécifique et la sérologie hydatique est souvent négative car les localisations parasitaires au niveau du système nerveux central n’induisent que difficilement des anticorps sériques. Le traitement du kyste hydatique cérébral est chirurgical [2, 3] (dans notre série, tous nos patients ont été opérés) ; son but est d’exciser l’ensemble du kyste sans provoquer sa rupture afin d’éviter la dissémination des scolex, le risque de récidives et de réactions anaphylactiques qui seraient responsables de collapsus circulatoire et d’arrêt cardiaque, garantissant ainsi une guérison définitive [1, 3]. La technique chirurgicale la plus utilisée est celle décrite par Arana Iniguez et consiste en l’accouchement du kyste par hydro-dissection utilisant le sérum salé hypertonique. Alors que l’hydatidose cérébrale reste considérée par la plupart des auteurs comme une maladie bénigne, des séquelles fonctionnelles motrices et/ou visuelles et des complications postopératoires à type d’hématome sous dural ou de méningite bactérienne peuvent survenir, et ce d’autant que les kystes hydatiques sont multiples et/ou récidivants [**3**]. Aucun cas de récidive n’a été noté dans notre série et tous nos patients avaient une bonne évolution clinique.

## Conclusion

L’hydatidose cérébrale est une affection rare qui touche essentiellement l’enfant. Grâce à un aspect pathognomonique, la TDM permet dans la majorité des cas un diagnostic préopératoire de certitude. L’IRM offre cependant une meilleure délimitation topographique notamment dans les localisations multiples. Elle aide au choix de la voie d’abord chirurgicale permettant la prise des mesures nécessaires pour éviter la dissémination peropératoire de liquide et/ou de vésicules hydatiques. L’apport des séquences Flair et de diffusion devient capital dès que le kyste est remanié et la spectroscopie pourrait constituer également un complément diagnostique très utile en cas de diagnostic différentiel. Le pronostic est généralement bon si le diagnostic est fait rapidement menant à un traitement précoce permettant par conséquent d’éviter les séquelles neurologiques.

### Etat des connaissances actuelles sur le sujet

- L’hydatidose cérébrale est une affection rare qui touche essentiellement l’enfant;
- Les signes cliniques sont ceux d'un processus intracrânien;
- La TDM permet dans la majorité des cas un diagnostic préopératoire de certitude.

### Contribution de notre étude à la connaissance

- L’intérêt de l’IRM offrant une meilleure délimitation topographique notamment dans les localisations multiples: elle aide au choix de la voie d’abord chirurgicale permettant la prise des mesures nécessaires pour éviter la dissémination peropératoire de liquide et/ou de vésicules hydatiques;
- L’apport des séquences Flair et de diffusion devient capital dès que le kyste est remanié et la spectroscopie pourrait constituer également un complément diagnostique très utile en cas de diagnostic différentiel;
- Le pronostic est généralement bon si le diagnostic est fait rapidement menant à un traitement précoce permettant par conséquent d’éviter les séquelles neurologiques.
